# Public Health Workforce training Laboratorium: Pilot e-Learning course on CBE and PBL models: Guglielmo Arzilli

**DOI:** 10.1093/eurpub/ckac131.091

**Published:** 2022-10-25

**Authors:** D Barbina, F Riccardo, A Di Pucchio, M Del Manso, M Mammoli, R Croci, A Vittozzi, A Mazzaccara, R Ferrelli, S Brusaferro

**Affiliations:** 1 Training Office, National Institute of Health, Rome, Italy; 2 Department of Infectious Desaese, National Institute of Health, Rome, Italy; 3 Information Technology, National Institute of Health, Rome, Italy; 4 School of Medicine, University Vita-Salute San Raffaele, Milan, Italy; 5 National Institute of Health, Rome, Italy

## Abstract

**Background:**

The National Institute of health (ISS), within the G20 Health Working Group, launched at March 2021 an innovative training Laboratorium, to strengthen the capacity and competencies of the Public Health Workforce (PHW) on prevention, preparedness and response to health crises. It was recognised in the G20 Declarations of Ministers of Health and Leaders and it was aimed to the development of training tools suitable for distance learning. ISS proposed the design of e-Learning courses based on an integration of WHO’s Competency-Based Education (CBE) and Problem Based Learning (PBL) models.

**Objectives:**

Describe the design of a pilot e-Learning course based on CBE- PBL models, focused on Epidemic Intelligence (EI).

**Results:**

The pilot e-Learning course “Use of Epidemic Intelligence systems with a particular focus on event-based surveillance for pandemic preparedness” will be delivered by ISS e-Learning platform https://www.eduiss.it. By the end of the course participants will be able to evaluate the potential use/applicability of EI systems, focusing on event-based surveillance for preparedness and early warning at country/institutional level. CBE is the basis for articulating the outcomes and identifying the required competencies and PBL for creating learning activities associated to learning outcomes. Interactive tools (exercise, web pages, quiz and other activities) are associated to learning objectives. The course is offered to public health professionals free of charge, in English language. About 16 hours are required to complete all the activities and receive attendance certification.

**Conclusions:**

Continuously updated training of PHW is a hallmark to better face health challenges. In our proposal, the learning methodology integrates CBE vision with PBL approach, adapting both to e-Learning context.

**Aknowledement:**

Pietro Carbone, Debora Guerrera, Federica Regini, Licia Bacciocchi, Stefania Bocci, Silvia Stacchini, Giovanna Failla - ISS, Rome Italy

**Key messages:**

• Design an innovative e-learning course through synergic integration of two educational models crucial in public health, CBE and PBL.

• Adapt e valorize CBE and PBL models in the context of e-Learning.

